# Application of intravitreal aflibercept to treat bilateral exudative retinal detachment secondary to retinitis pigmentosa: Case report and review of literature

**DOI:** 10.1097/MD.0000000000036589

**Published:** 2023-12-22

**Authors:** Chia-Chen Kao, Kuo-Jen Chen, Kai-Chun Cheng

**Affiliations:** a Department of Ophthalmology, Kaohsiung Medical University Hospital, Kaohsiung, Taiwan; b Department of Ophthalmology, School of Medicine, College of Medicine, Kaohsiung Medical University, Kaohsiung, Taiwan; c Department of Ophthalmology, Kaohsiung Municipal Siaogang Hospital, Kaohsiung, Taiwan.

**Keywords:** aflibercept, anti-VEGF agents, exudative retinal detachment, intravitreal injection, retinitis pigmentosa (RP)

## Abstract

**Rationale:**

Exudative retinal detachment with macular edema is one of the complications of retinitis pigmentosa (RP). In this report, we present a case who treated with intravitreal injection of anti-vascular endothelial growth factor (anti-VEGF) in RP-related exudative retinal detachment and subsequently improved with favorable outcome.

**Patient Concern:**

A 49-year-old man, with a history of RP, had persistent blurred vision and was newly diagnosed with bilateral shallow exudative retinal detachment and macular edema.

**Diagnosis:**

Fluorescein angiography showed bilateral diffuse dye leakage with macular pooling, and systemic survey excluded the possibility of infection or autoimmune disease.

**Interventions:**

The patient was treated with intravitreal injection of aflibercept, one of the anti-VEGF agents, for bilateral eyes. Recurrent exudative retinal detachment and macular edema were noted, and repeated intravitreal injections of aflibercept in bilateral eyes were then arranged. Subsequently, bilateral macular edema and exudative retinal detachment subsided again, and the treatment course lasted for approximately 1 year.

**Outcomes:**

After 1 year, the exudative retinal detachment with macular edema was much improved. In the meanwhile, visual functional improvement was also achieved.

**Lessons:**

This case illustrated the possibility of intravitreal injection of anti-VEGF therapy for the treatment of this rare complication of RP, and it may be a newly explored alternative treatment.

## 1. Introduction

In recent literature of retinitis pigmentosa (RP), cryotherapy and laser photocoagulation were reported as treatment for Coats’-like serous retinal detachment. However, we presented a case with RP-related bilateral shallow exudative retinal detachment and macular edema, which showed different fundus pattern from previous reports. In addition, in our case, anti-VEGF (vascular endothelial growth factor) therapy was an effective treatment in this rare complication of RP.

## 2. Case report

**Figure 1.**
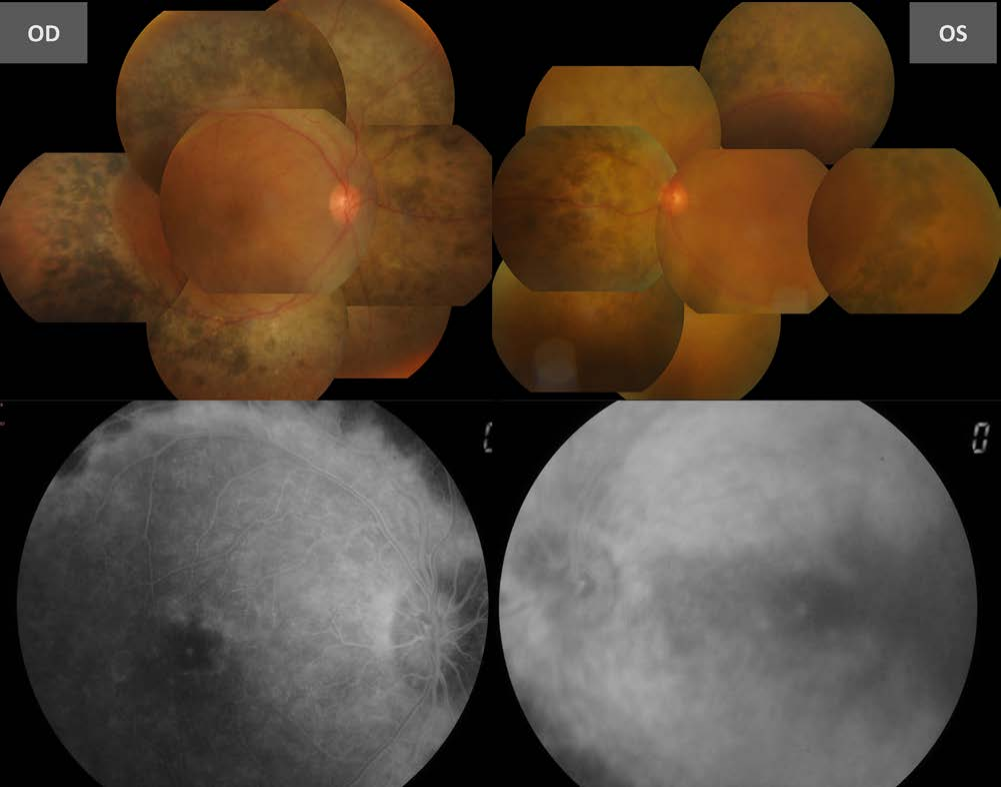
Color fundus revealed shallow exudative retinal detachment with macular edema in bilateral eyes. FAG showed bilateral diffuse dye leakage with macular pooling. FAG = fluorescein angiography.

**Figure 2.**
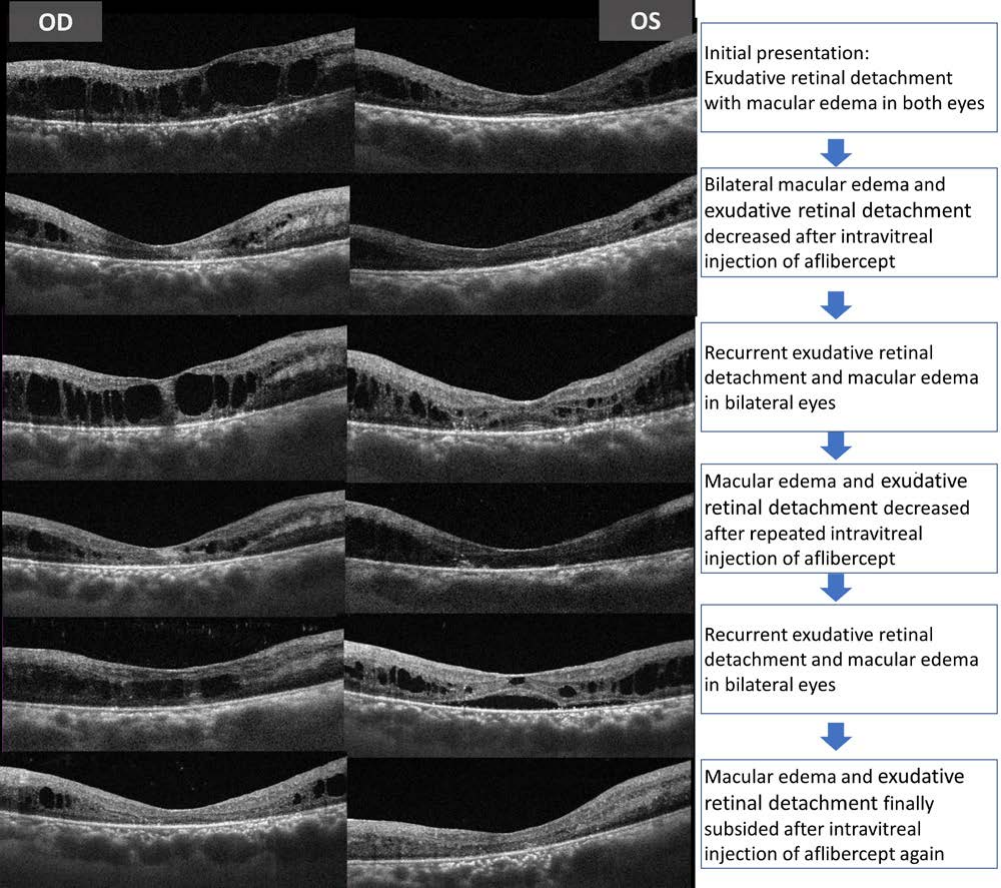
OCT showed resolution of bilateral macular edema and exudative retinal detachment after repeated intravitreal injection of aflibercept. OCT = optical coherence tomography.

The 49-year-old man had a history of RP. He received cataract surgery for the right eye due to progressive blurred vision. In the following 2 months, he still felt persistent bilateral blurred vision. Visual acuity (VA) was counting fingers 30cm in the right eye and 20/400 in the left eye. Slit lamp examination showed silent anterior chamber in bilateral eyes, posterior chamber intraocular lens with mild after-cataract in the right eye, and nuclear sclerosis with posterior subcapsular opacity in the left eye. The fundus examination showed bilateral mid-peripheral retinal pigmentary change and newly appeared diffuse shallow exudative retinal detachment with posterior pole and peripheral retinal involvement (Fig. 1). Optical Coherence Tomography revealed marked macular edema with shallow exudative retinal detachment in bilateral eyes (Fig. 2), and fluorescein angiography showed bilateral diffuse dye leakage with macular pooling (Fig. 1). Systemic survey excluded the possibility of infection, and autoimmune disease was not favored due to normal limits for rheumatoid factor, antinuclear antibody and HLA-B27.

Afterwards, intravitreal injection of 2 mg aflibercept was performed for bilateral eyes, and macular edema resolution with decreased exudative retinal detachment was found. The patient subjectively felt improving vision, and his VA also improved to 20/600 in the right eye and 20/200 in the left eye. However, recurrent macular edema and exudative retinal detachment were then noted in the following months. Repeated intravitreal injections of aflibercept in bilateral eyes were then arranged, and subsequently, bilateral macular edema and exudative retinal detachment subsided again (Fig. 2). The treatment course lasted for approximately 1 year, and the final VA was 20/600 in the right eye and 20/40 in the left eye. Both eyes achieved visual functional improvement, and the condition was stable until now.

## 3. Discussion

RP may be complicated with choroidal neovascularization (CNV), cystoid macular edema, or exudative retinal detachment in the late stage in rare circumstances. VA may also gradually worsen due to RP-related macular atrophic degeneration or optic neuropathy, and some patients might develop neovascular glaucoma with progression to ocular phthisis.^[1]^

As for RP-related retinal detachment, it is associated with a spectrum of exudative, tractional, and rhegmatogenous retinal detachment. Exudative retinal detachment does not usually require surgical repair, although cryotherapy or laser are reported occasionally necessary in sight-threatened patients in certain condition. In previous literature reports, RP with exudative subretinal fluid might present with bilateral Coats’-like serous retinal detachment, including telangiectasia and prominent lipid deposition, and such lesions often localize inferiorly and require laser photocoagulation.^[2]^ In comparison, our patient had bilateral diffuse shallow exudative retinal detachment and macular edema, without appearance of telangiectasia or lipid deposits, and the lesion was located from macula to peripheral retina. The mechanism of conventional laser photocoagulation and cryotherapy is possibly to damage the retinal pigment epithelium and choriocapillaris, and this can result in permanent choroidal and retinal atrophy. Laser photocoagulation may also transiently worsen macula edema and exudative subretinal fluid. Therefore, in our case with diffuse retina detachment, it would be difficult to do laser photocoagulation for the diffuse subretinal fluid, and it could be unsafe to perform cryotherapy for retinal lesions in posterior pole. In addition, topical dorzolamide was demonstrated to be effective for the treatment of cystoid macular edema in patients with RP,^[3]^ but there was no reported of positive treatment effect for RP related exudative retinal detachment.

Apart from the above consideration, the pathophysiology and mechanism of exudative retinal detachment in our patient might be different from the previous reports of Coats’-like pattern serous retinal detachment. In our case, there could be the possibility of occult neovascular lesions or vascular permeability increase, leading to diffuse fluorescein leakage and dye pooling in fluorescein angiography. Intravitreal injection of anti-VEGF is considered to reduce the choroidal and retinal vascular permeability, to stabilize the blood–retinal barrier and thereby to inhibit exudation into retinal space. Thus, intravitreal injection of aflibercept was effective for our patient, and exudative retinal detachment and macular edema subsided after repeated anti-VEGF injection; however, only one paper mentioned this treatment strategy in Coats’-like exudative vitreoretinopathy in RP,^[4]^ so further reports are required to help ophthalmologists deal with this rare situation.

Although there were previously concerns regarding long-term inhibition of VEGF, leading to inhibited neuro-protective effect, anti-VEGF agent is recently viewed as a safe management for neovascularization and macular edema secondary to RP.^[5]^ Occasionally, CNV may be successfully managed with a single intravitreal anti-VEGF injection, but most cases require more than a one-year period to achieve stabilization in RP-related CNV.^[6]^ In our case, anti-VEGF agents were also effective in exudative RD and macular edema, but there might be recurrence in the following months. Our patient received repeated intravitreal injections of aflibercept in both eyes for 3 times during 1 year, and the exudative retinal detachment with macular edema subsided and maintained stable condition. In the meanwhile, the patient also achieved improvement of VA.

In conclusion, heightened awareness of potential complications of RP might assist earlier diagnosis, thus facilitating timely intervention leading to better prognosis. Consequently, detailed fundus examination should be performed in RP patients with deteriorated vision; additionally, RP related exudative retinal detachment and macular edema could indicate occult neovascular lesions or increased vascular permeability, and anti-VEGF therapy could be considered as an alternative therapeutic option in this rare condition.

## Author contributions

**Conceptualization:** Chia-Chen Kao.

**Investigation:** Chia-Chen Kao.

**Methodology:** Chia-Chen Kao.

**Software:** Kuo-Jen Chen.

**Supervision:** Kai-Chun Cheng.

**Validation:** Chia-Chen Kao, Kuo-Jen Chen.

**Writing – original draft:** Chia-Chen Kao.

**Writing – review & editing:** Kai-Chun Cheng.

## References

[1] Chan WO Brennan N Webster AR. Retinal detachment in retinitis pigmentosa. BMJ Open Ophthalmol. 2020;5:e000454. 10.1136/bmjophth-2020-000454 PMC7351280 32671228

[2] Kan E Yilmaz T Aydemir O. Coats-like retinitis pigmentosa: reports of three cases. Clin Ophthalmol. 2007;1:193–8. 19668510 PMC2704518

[3] Ikeda Y Hisatomi T Yoshida N. The clinical efficacy of a topical dorzolamide in the management of cystoid macular edema in patients with retinitis pigmentosa. Graefes Arch Clin Exp Ophthalmol. 2012;250:809–14. 22215259 10.1007/s00417-011-1904-5

[4] Moinuddin O Sathrasala S Jayasundera KT. Coats-like exudative vitreoretinopathy in retinitis pigmentosa: ocular manifestations and treatment outcomes. Ophthalmol Retina. 2021;5:86–96. 32507488 10.1016/j.oret.2020.03.026 PMC8086515

[5] Manabu M Akio O Maho O. Long-term efficacy and safety of anti-VEGF therapy in retinitis pigmentosa: a case report. BMC Ophthalmol. 2018;18:248. 30217183 10.1186/s12886-018-0914-z PMC6137720

[6] Sayadi J Miere A Souied EH. Type 3 neovascularization associated with retinitis pigmentosa. Case Rep Ophthalmol. 2017;8:245–9. 28512428 10.1159/000471790 PMC5422741

